# Factors Influencing Non-adherence to Treatment Among New Patients With Tuberculosis in the Field Practice Area of a Tertiary Care Hospital in Hyderabad: A Cross-Sectional Study

**DOI:** 10.7759/cureus.107370

**Published:** 2026-04-19

**Authors:** Durga Anbalagan, Sandeep Kumar Dharnamoni, Prasanth Kumar Sarella, Lakshman Rao RL, Adarsh Kumar K

**Affiliations:** 1 Department of Community Medicine, Employees State Insurance Corporation Medical College and Hospital, Hyderabad, Hyderabad, IND; 2 Department of Community Medicine, Government Medical College, Maheshwaram, Maheshwaram, IND; 3 Department of Community Medicine, Government Medical College, Rajamahendravaram, Rajamahendravaram, IND; 4 Department of Community Medicine, Government Medical College, Yadadri, Yadadri, IND; 5 Department of Otorhinolaryngology, Yashoda Hospital, Hyderabad, IND

**Keywords:** drug compliance, drug-resistant tb, poor adherence, tuberculosis, tuberculosis treatment adherence

## Abstract

Introduction: Tuberculosis (TB) is an infectious disease caused by *Mycobacterium tuberculosis *(MT), which primarily affects the lungs (pulmonary tuberculosis) but can also involve other organs, such as bone, lymph nodes, brain, and kidney (extrapulmonary tuberculosis). Although it is a curable and preventable disease, it continues to be a major public health problem. Adherence to therapy plays an important role in treatment outcome. The objectives are to study the prevalence of non-adherence to anti-tuberculosis treatment among new patients with pulmonary tuberculosis in the field practice area of a tertiary care hospital in Hyderabad and to determine the factors influencing non-adherence to treatment among new patients with pulmonary tuberculosis.

Methodology: A community-based cross-sectional study was carried out in the field practice areas of Osmania Medical College, Hyderabad, between October 2020 and March 2021. All newly diagnosed patients with pulmonary tuberculosis registered under Directly Observed Treatment Short-Course (DOTS) from October 2020 to March 2021 were taken into the study. Taking the prevalence of non-adherence to anti-tuberculosis treatment as 50% and absolute error as 5% with 95% confidence interval, the sample size was calculated as 385, and the participants were chosen by simple random sampling. A predesigned, pretested, and semi-structured questionnaire was used for collecting data on sociodemographic and socioeconomic factors, clinical characteristics, and factors influencing non-adherence to treatment, such as patient-related factors, health system-related factors, stigma/discrimination, disease-related factors, and medicine-related factors, by interviewing the patients.

Results: The mean age of the study population was 43 years. Of the patients, 77.7% (299) were men and 22.3% (86) were women. In this study, 19.48% of the study participants had non-adherence to treatment. Socioeconomic status, educational status, marital status, side effects of drugs, awareness about treatment completion, and smoking were significantly associated with non-adherence to treatment.

Conclusion: Marital status, educational status, smoking, and socioeconomic constraints contribute to poor adherence. Improving adherence to treatment requires a comprehensive, patient-centered approach that combines health education, counselling, social support, and strengthened healthcare delivery systems.

## Introduction

Tuberculosis (TB) is a major public health concern globally. It is an infectious disease caused by *Mycobacterium tuberculosis* (MTB), which is transmitted through the air by droplet nuclei or ingesting infected milk or meat (bovine TB) [1]. Tuberculosis is both preventable and curable. People with pulmonary tuberculosis (TB disease in the lungs) can infect others through droplet nuclei when they cough, sneeze, or talk [1]. With early detection and prompt treatment, people with the disease eventually get cured. For the same reason, the focus of public health machinery is on tuberculosis. Treatment adherence is one of the key factors affecting the outcome of the therapy. The impact of Directly Observed Treatment Short-Course (DOTS) in reducing the TB incidence has been hindered by non-adherence to treatment. Assessing the factors influencing treatment adherence will give the reasons behind high default rates and low treatment success rates.

Every year, millions of people are affected by tuberculosis. Despite being preventable and curable, 1.5 million people die from tuberculosis every year, making it the world’s top infectious killer [2]. India accounts for a quarter of the global TB burden, and in 2018, the estimated TB incidence was 2.69 million (199/100,000 population) [2]. In India, the incidence rate had reduced from 237 per lakh population in 2015 to 199 per lakh population in 2022. The mortality rate had also declined from 28 per lakh population in 2015 to 23 per lakh population in 2022 [3]. According to the World Health Organization (WHO), elimination means that there should be fewer than one person with TB for a population of a million people [4]. A significant amount of work remains to be done in order to achieve elimination by 2030.

This study is one of the very few studies conducted to identify factors associated with non-adherence to tuberculosis treatment in Hyderabad. Identifying the factors influencing non-adherence to TB treatment will contribute to the existing body of knowledge and also provide insights into the factors contributing to high default rates and low treatment success rates. Once the factors influencing non-adherence to treatment are identified, then targeted strategies can be formulated to address them. This study will benefit patients with tuberculosis as the findings may be used to formulate strategies and guidelines to improve the quality of care, thereby improving treatment outcomes.

The objectives are to study the prevalence of non-adherence to anti-tuberculosis treatment among new patients with pulmonary tuberculosis in the field practice area of a tertiary care hospital in Hyderabad and to determine the factors influencing non-adherence to treatment among new patients with pulmonary tuberculosis.

## Materials and methods

The present study is a community-based cross-sectional study carried out in the field practice areas of Harraj Penta (urban field practice area) and Patancheru (rural field practice area) of the Department of Community Medicine, Osmania Medical College, Hyderabad, between October 2020 and March 2021. All newly diagnosed patients with pulmonary tuberculosis registered under DOTS from October 2020 to March 2021 and have given consent to participate were taken into the study. Patients with tuberculosis who did not give informed consent, were unable to communicate, and were seriously ill at the time of data collection, and those with HIV co-infection were excluded from the study.

Operational definition

Non-adherence

Missing ≥2 consecutive weeks of DOTS was considered as non-adherence [5].

Taking the prevalence of non-adherence to anti-tuberculosis treatment as 50% [6], with an absolute error of 5% with 95% confidence interval, the sample size was calculated as 385. The study participants were recruited into the study using simple random sampling. There are 26 DOTS centers under the Osmania Tuberculosis Unit (TU) and 1 in the rural field practice area. By identifying the DOTS centers under urban and rural field practice areas, the list of information containing new patients with pulmonary tuberculosis who were under treatment was obtained. From the list of 783 patients, a sample of 385 patients were selected randomly by generating the random numbers using Microsoft Excel (Microsoft Corp., Redmond, WA). The study subjects who satisfied the inclusion and exclusion criteria were selected for the study.

A pilot study was conducted among 39 study subjects by taking 10% of the sample size in the field practice area, and the questionnaire was pretested and validated. A predesigned, pretested, and semi-structured questionnaire was used for collecting data on sociodemographic and socioeconomic factors, clinical characteristics, and factors influencing non-adherence to treatment, such as patient-related factors, health system-related factors, stigma/discrimination, disease-related factors, and medicine-related factors, by interviewing the patients. The modified BG Prasad classification was used to classify the socioeconomic status of the study subjects [7].

Once sampling was completed, a list of potential participants was compiled using their names and contact details, and data were collected through home visits. The participants were interviewed after reading the individual participant consent form that detailed the title and purpose of the study, as well as the rights of the participant. Whenever a participant has given consent to be interviewed, he/she was asked to provide a written consent by signing or fingerprinting.

Data was entered on spreadsheets using Microsoft Excel and analyzed using Epi Info version 7.2.2.6. Analysis included descriptive summary statistics and graphical summaries in the form of charts (pie, bar, and cross-tabulations). Univariate analysis using the chi-square test with a significance level at 5% was used, and multivariate logistic regression analysis was performed to determine the association of various independent variables with the dependent variable after controlling for the effect of confounding factors using SPSS (IBM Corp., Armonk, NY). p<0.05 was considered statistically significant. Multicollinearity was tested using the variance inflation factor. Nagelkerke R-square was 0.397, which meant that 39.7% of the variation in the outcome was explained by the variables of interest. The Hosmer-Lemeshow test was statistically not found to be significant, which meant that the model was fit to predict outcomes.

Ethical clearance was obtained from the Institutional Ethics Committee of Osmania Medical College, Koti, Hyderabad (approval number: ECR/300/Inst/AP/2013/RR-16).

## Results

**Figure 1:**
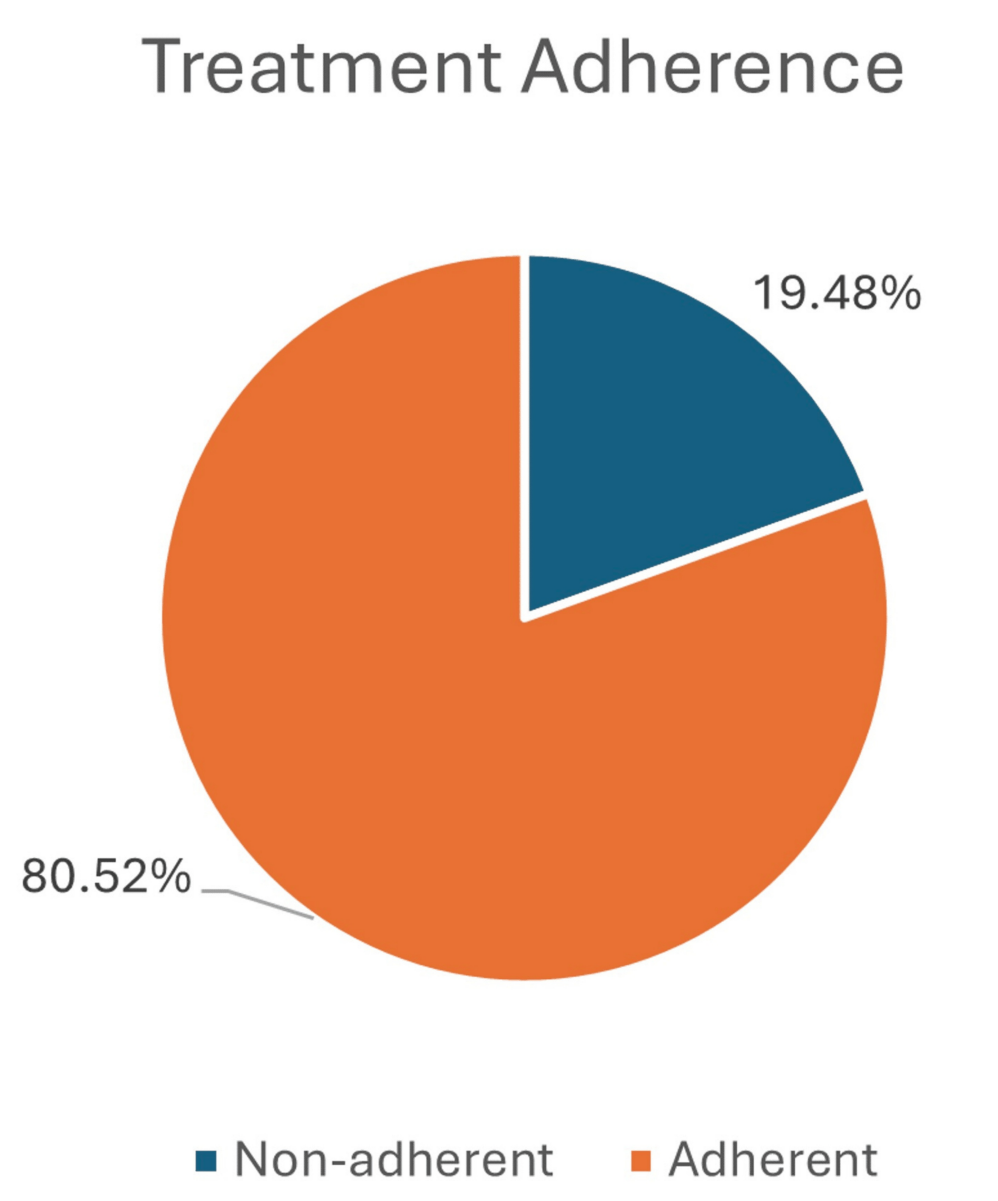
Distribution of study subjects based on treatment adherence

The mean age of the study population was 43 years. The majority (75.84% (292)) of the study subjects belong to the 19-60-year age group. Of the study subjects, 5.20% were below 18 years of age. Of the patients, 77.7% (299) were men and 22.3% (86) were women. In this study, 19.48% of the study participants had non-adherence to treatment (Figure 1).

Table 1 shows the knowledge regarding tuberculosis among the study participants.

**Table 1:** Knowledge regarding tuberculosis among the study participants

Knowledge variables		Adherent (number)	Non-adherent (number)
TB is a serious disease.	Yes	230	30
	No	80	45
TB spreads by droplets.	Yes	297	55
	No	13	20
Cough with sputum for >2 weeks or sputum with blood is a criterion for suspecting TB.	Yes	283	42
	No	27	33
For TB, treatment is given in TB treatment centers.	Yes	295	58
	No	15	17
TB can be cured by taking medications regularly.	Yes	297	60
	No	13	15
Can you stop taking medications once your symptoms disappear?	Yes	19	26
	No	309	49
Do you know the duration of TB treatment?	Yes	297	57
	No	13	18
Do you know about the consequences of incomplete TB treatment?	Yes	282	33
	No	28	42
Vaccination can prevent TB.	Yes	266	19
	No	44	56
Proper hygiene, nutrition, and a healthy lifestyle can prevent TB.	Yes	294	50
	No	16	25

Table 2 shows the factors influencing non-adherence to treatment among the study participants. There was a statistically significant association between marital status, socioeconomic status, education, side effects of drugs, awareness about treatment completion, improvement of symptoms, loss of daily wages, stigma/discrimination faced, and smoking, and treatment adherence.

**Table 2:** Factors influencing non-adherence to treatment p<0.05 was considered significant.

Variable		Adherent	Non-adherent	Total	p-value
Age	<18 years	19	1	20	0.159
	19-59 years	230	62	292	
	>60 years	61	12	73	
Gender	Male	240	59	299	0.937
	Female	70	16	86	
Religion	Hindu	132	31	163	0.216
	Christian	25	2	27	
	Muslim	153	42	195	
Marital status	Single	94	12	106	0.044
	Married	168	49	217	
	Widowed	36	8	44	
	Separated/divorced	12	6	18	
Type of family	Nuclear	196	54	250	0.356
	Joint	51	9	60	
	3 generation	63	12	75	
Educational status	Literate	194	19	213	<0.001
	Illiterate	116	56	172	
Socioeconomic class	I - upper	11	2	13	0.032
	II - upper middle	47	8	55	
	III - middle	146	49	195	
	IV - lower middle	77	15	92	
	V - lower	29	1	30	
Awareness about the disease condition	Yes	304	70	374	0.068
	No	6	5	11	
Forgetfulness	Yes	76	24	100	0.238
	No	234	51	285	
Side effects	Yes	301	67	368	0.008
	No	9	8	17	
Change of residence	Yes	15	7	22	0.220
	No	295	68	363	
Travel time > 15 minutes	Yes	208	56	264	0.260
	No	102	19	121	
Transport difficulty	Yes	34	10	44	0.707
	No	276	65	341	
Improvement of symptoms	Yes	285	61	346	0.011
	No	25	14	39	
Awareness about treatment completion	Yes	295	64	359	0.005
	No	15	11	26	
Family support	Yes	278	62	340	0.134
	No	32	13	45	
Stigma/discrimination faced	Yes	212	61	273	0.038
	No	98	14	112	
Job security	Yes	202	54	256	0.322
	No	108	21	129	
Loss of daily wages	Yes	276	59	335	0.027
	No	34	16	50	
Longer duration of treatment	Yes	232	49	281	0.128
	No	78	26	104	
Left hometown for other reasons	Yes	18	7	25	0.394
	No	292	68	360	
Smoking	Yes	128	48	176	<0.001
	No	182	27	209	
Alcohol	Yes	94	32	126	0.061
	No	216	43	259	

Table 3 shows the multivariate logistic regression analysis for predictors of treatment adherence. Marital status, educational status, and smoking were significantly associated with treatment adherence. Only a few variables were entered in the final model of binary logistic regression. Non-adherence to treatment was 2.304 times higher among married individuals compared to those who were not married/separated or divorced. Similarly, those who were illiterate were found to have non-adherence 6.789 times more compared with those who were literate, and it was statistically significant. Regarding smoking, smokers had 2.283 times more non-adherence compared with non-smokers, which was also statistically significant.

**Table 3:** Multivariate logistic regression analysis for predictors of treatment adherence p<0.05 was considered significant. CI: confidence interval

Factors		Odds ratio	95% CI	p-value
Marital status	Single/separated	1		0.007
	Married	2.304	1.260-4.214	
Educational status	Literate	1		<0.001
	Illiterate	6.789	3.218-14.323	
Socioeconomic class	Upper	1		0.187
	Middle/lower	1.692	0.774-3.701	
Side effects	No	1		0.502
	Yes	0.562	0.105-3.023	
Awareness about treatment completion	Yes	1		0.597
	No	0.600	0.090-3.986	
Smoking	No	1		0.034
	Yes	2.283	1.064-4.896	

## Discussion

Poor adherence contributes to the worsening of tuberculosis by increasing drug resistance. Evidence from the literature shows that there are many factors influencing adherence to treatment among patients with tuberculosis.

In this study, the prevalence of non-adherence to anti-tuberculosis medications was 19.48% among the total study population. Similar findings were observed in a study done by Bagchi et al. [8] in Mumbai, where 16% (87) were non-adherent. In a study done by Bam et al. [9] in Nepal, 16.12% (25) were non-adherent. Noncompliance with treatment among patients with tuberculosis affects the control of the disease, thereby leading to increased burden of the disease and mortality [10].

It was observed that 77.7% of the study participants were men and 22.3% were women. The study showed higher non-adherence among men. This might be due to the fact that men are more likely to have physically demanding jobs, and loss of wages can be a significant problem. However, the difference in the prevalence of treatment adherence associated with the gender of the patients is not statistically significant. Similar findings were seen in the study done by Shahrezaei et al. [11] and Kulkarni et al. [6]. In contrast to this, the study conducted by Gohel et al. [12] showed higher non-adherence among women.

The present study reveals that most of the patients belong to the age group 19-60 years (75.84%). It was found that non-adherence to treatment was more common among the productive age group, which was not statistically significant. These findings were similar to those by Gohel et al. [12]. Another study conducted by Mittal et al. [13] showed that non-adherence to treatment was found to be more common among elderly patients, whereas very good compliance to treatment was observed among pediatric patients.

In the present study, among the total study population, most of the patients (50.64% (195)) belonged to the middle socioeconomic class. There was a significant association between treatment adherence and socioeconomic class (p=0.032). The difference in the prevalence of treatment adherence associated with loss of daily wages faced by the patients is also statistically significant (p=0.027). Similar findings were found in the study conducted by Krasniqi et al. [14], and the study found that some patients faced high transportation costs when traveling to the health facility.

In the present study, 45.7% were smokers. There is a strong association between treatment adherence and smoking (p=0.0006). The study by Khan et al. [15] showed that smoking has a strong influence on tuberculosis and is a major barrier to treatment success. Hence, the study indicates that smoking cessation is an effective way to decrease treatment failure and thereby drug resistance.

In the present study, forgetfulness was one of the reasons for missing the doses, although the difference in the prevalence of treatment adherence associated with forgetfulness of the patients is statistically not significant.

In the present study, the perceived feeling of wellness or feeling cured was one of the reasons reported by patients for their intention to discontinue their treatment. The difference in the prevalence of treatment adherence associated with improvement of symptoms is statistically significant (p=0.011). Often, patients are very ill when they begin treatment. However, with time, as treatment progresses, their condition improves, and symptoms begin to subside. This itself can become a barrier to continuing treatment, as patients may not feel the need to complete it once they begin to feel better [16]. There is a need to address this issue. Health education must be given to all patients to emphasize the importance of adherence regardless of perceived recovery, as this could have important implications for preventing treatment interruption and thereby drug resistance.

Patients’ knowledge about their disease and its treatment will enhance treatment adherence. It was found that the majority of the patients lack adequate knowledge regarding tuberculosis and are not aware of the actual cause, mode of transmission, and duration of treatment. Treatment literacy refers to providing accurate information about the disease and its treatment, enabling patients to take greater responsibility for their own care and to advocate for their rights when appropriate care is not provided [13]. According to the study by Dewalt et al. [17], lack of treatment literacy will have an impact on health outcomes, and treatment literacy improves compliance and thereby outcomes. Although this study did not analyze the concept of treatment literacy, knowledge regarding the disease was observed, which was inadequate.

Several respondents did not know the conventional treatment duration or the risk when stopping the treatment. This was consistent with the study findings of Khan et al. [18] and Wares et al. [19]. The patients’ knowledge regarding tuberculosis symptoms was good, as the majority of the study participants were able to identify that a cough with sputum for more than two weeks or sputum with blood is a criterion for suspecting tuberculosis. Knowledge regarding treatment duration was statistically significant, as those who did not know the duration of tuberculosis treatment were more likely to be non-adherent. Lack of health education may result in non-adherence, as patients will not complete the treatment. Failure to complete the treatment is often associated with developing resistance to the drugs. In the study by Bam et al. [9], in Nepal, it was found that 48% of non-compliant patients with tuberculosis discontinued treatment once they felt better and symptom-free, believing that the disease was cured. Other studies, such as those done by O’Boyle et al. [20], Pushpananthan et al. [21], and Peltzer et al. [22], also had similar findings about feeling better being associated with non-adherence to treatment.

Most patients in this study reported TB medication side effects. Only a few had said they did not experience any side effects. Most of the patients further said the side effects disappeared after their adaptation to the medication. The difference in the prevalence of treatment adherence associated with side effects of the treatment is statistically significant (p=0.008). The side effects of the drugs can discourage patients from continuing treatment, especially during the initial weeks when they may feel worse. Therefore, it is important to provide health education to all patients and to manage any side effects promptly as they arise.

Similar findings were found in the studies conducted by Janakan and Seneviratne [23] and Okanurak et al. [24], which showed medicine side effects as factors affecting treatment adherence. The study by Janakan and Seneviratne [23] also indicated that delays in addressing adverse effects or dismissing patients’ complaints about medication can lead to treatment non-adherence.

According to WHO [25], most patients with tuberculosis complete treatment without experiencing significant side effects from the anti-tuberculosis medications. Common side effects that patients typically experience include skin rashes, visual and auditory disturbances, burning sensations in the limbs, and limb pain. These side effects may cause patients to stop the treatment. Hence, early diagnosis and prompt treatment of the side effects should be done immediately.

In the present study, the health facilities were located close to the majority of the interviewed patients’ residences. Very few patients reported a change of residence during their treatment course. The difference in the prevalence of treatment adherence associated with change of residence of the patients, travel time to the treatment center, and facing transport difficulty is statistically not significant (p>0.05). Most of the patients were supervised by a family member while taking the treatment. Several studies, such as those by Bam et al. [26], Hasker et al. [27], O’Boyle et al. [20], Pushpananthan et al. [21], and Needham et al. [28], showed that transportation costs contribute to treatment non-adherence, particularly once patients start feeling symptomatically better.

Most participants experienced societal stigma and discrimination in this study. The difference in the prevalence of treatment adherence associated with stigma/discrimination faced by the patients in society is statistically significant (p=0.038). Perceived stigma or discrimination in the community may act as a barrier, preventing patients from disclosing their disease to family or community members. Furber et al. [29] concluded that stigma and discrimination toward patients with tuberculosis and HIV lead to delays in seeking healthcare, testing, and treatment initiation, ultimately resulting in poor health outcomes.

Multivariate logistic regression analysis for predictors of treatment adherence is shown in Table 3. Marital status, educational status, and smoking were significantly associated with treatment adherence. Only a few variables were entered in the final model of binary logistic regression. Non-adherence to treatment was 2.304 times higher among married individuals compared to those who were not married/separated or divorced. Similarly, those who were illiterate were found to have non-adherence 6.789 times more compared with those who were literate, which was statistically significant. Regarding smoking, smokers had 2.283 times more non-adherence compared with non-smokers, which was also statistically significant.

The present study was conducted with a small sample size, which consisted of patients with tuberculosis who were on treatment from only selected areas of Hyderabad. Therefore, the findings of this study may not be generalizable. During the interview, the responses given by the study subjects might not have been accurate due to recall bias. Another limitation was the short time span, which did not allow follow-up of the study subjects to know the treatment outcome.

## Conclusions

Poor adherence to tuberculosis treatment remains a major barrier to effective disease control. It leads to prolonged infectiousness, increased transmission in the community, higher rates of relapse, and the emergence of drug-resistant tuberculosis. Factors contributing to poor adherence include long treatment duration, adverse drug effects, lack of patient awareness, stigma, socioeconomic constraints, and inadequate health system support. Improving adherence to treatment requires a comprehensive, patient-centered approach that combines health education, counselling, social support, and strengthened healthcare delivery systems. Strategies such as Directly Observed Therapy (DOT), digital adherence technologies, and community-based interventions are essential to ensure treatment completion. Addressing these challenges is critical for achieving national and global TB elimination goals and reducing the burden of tuberculosis.
